# Hyaluronic Acid‐Recombinant Collagen Type III Electrospun Nanomembranes: A Stratum Corneum‐Navigated Cutaneous Delivery Platform for Skin Rejuvenation

**DOI:** 10.1111/jocd.71127

**Published:** 2026-08-17

**Authors:** Xinyu Peng, Songhua He, Xiaofeng Qiu, Zhanhua Fan, Qiudong Liao

**Affiliations:** ^1^ Nox Bellcow Cosmetics Co., Ltd. Zhongshan Guangdong China

**Keywords:** delivery/vectorization/penetration, electrospinning, nanomembrane, skin barrier, skin benefit

## Abstract

**Objective:**

This study tests the hypothesis that electrospun nanomembranes composed of recombinant human collagen type III (rhCol III, 35–50 kDa) and hyaluronic acid (HA, 20–40 kDa) enable stratum corneum–navigated cutaneous delivery of these two substances, thereby synergistically enhancing dermal physiology.

**Methods:**

RhCol III (35–50 kDa) and HA (20–40 kDa) were dissolved in ultrapure water (Milli‐Q, 18.2 MΩ·cm) at a mass ratio of 5.25:1 (w/w) to yield a 25% (w/v) total polymer concentration. Electrospinning was performed using an electrospinning device (YT‐MS‐III) at 8–9 kV voltage and a 15–18 cm tip‐to‐collector distance, yielding nanomembranes with fiber diameters of 187 ± 42 nm (*n* = 30). Control serum was prepared by dissolving rhCol III/HA powder in water; the composition and concentration are identical with the nanomembranes rehydrated by 7× the dry membrane weight (simulating clinical reconstitution). Ex vivo penetration was quantified using Franz diffusion cells (3 cm^2^ area) on porcine dorsal skin (TEWL < 15 g·m^−2^·h^−1^); HA was measured via HPLC (Agilent 1260, MCI GEL column, 1% phosphoric acid mobile phase, 232 nm detection); rhCol III was quantified by immunofluorescence using anti‐His‐tag antibody (Abcam ab9108, 1:500) and Alexa Fluor 488–conjugated secondary antibody (Thermo Fisher A11001, 1:1000). A randomized, split‐face, 4‐week clinical trial enrolled 35 healthy Asian volunteers (45 ± 1.0 years; wrinkle grade 2–4) to assess hydration (Corneometer CM825), barrier function (Tewameter TM300), elasticity (Cutometer MPA580), wrinkles (VISIA‐CR + Image‐Pro Plus 7.0), and erythema (Mexameter MX18).

**Results:**

At 24 h, rhCol III‐HA nanomembranes achieved a cumulative HA permeation of 0.136 ± 0.009 mg·cm^−2^ (mean ± SD), representing a 72.15% increase over control serum (0.079 ± 0.001 mg·cm^−2^; *p* < 0.001). Apparent permeability coefficient (K_p_) was 4.90 × 10^−3^ ± 1.2 × 10^−4^ mg·cm‐2·h^−1^ for nanomembranes vs. 2.50 × 10^−3^ ± 8.5 × 10^−5^ mg·cm‐2·h^−1^ for serum (*p* = 0.003). rhCol III immunofluorescence signal in the dermis was 3.86 ± 0.31‐fold higher in nanomembrane‐treated skin versus serum controls at 24 h (*p* < 0.001). Clinically, 4‐week electrospun nanomembrane treatment increased stratum corneum hydration by 47.82% (*p* < 0.001), reduced TEWL by 41.0% (*p* < 0.001), improved elasticity (R2) by 16.58% (*p* < 0.001), decreased periocular wrinkle area ratio by 36.88% (*p* = 0.001), and lowered erythema index by 20.34% (*p* < 0.001) vs. control (without treatment).

**Conclusion:**

Electrospun rhCol III‐HA nanomembranes function as a stratum corneum–navigated cutaneous delivery platform that overcomes classical molecular weight barriers (20–50 kDa), enabling co‐delivery of bioactive macromolecules (generally molecular weight > 10 kDa, e.g., rhCol III, HA) with statistically significant enhancements in skin hydration, barrier integrity, biomechanics, and appearance.

## 1 Introduction

Skin constitutes the largest organ within the human body. Its primary function is to offer a robust barrier that enables the body to fend off external intrusions, maintain its internal homeostasis, and resist ultraviolet radiation [1]. Consequently, the skin's barrier poses a challenge to the dermal delivery of drugs or active substances. There are three distinct routes through which a molecule can traverse the stratum corneum, namely the transcellular, intercellular, and appendageal (i.e., through the eccrine/sweat glands or hair follicles) routes [2, 3]. The appendageal route is regarded as relatively insignificant partly due to the relatively small surface area of the appendages [4]. Despite being longer than the transcellular route, the intercellular pathway is generally acknowledged as the main route [5]. The barrier function is mainly accomplished by the stratum corneum because of its lipophilic nature and the strong cohesion among the cornified cells [6]. Hence, numerous novel approaches have been employed to design transdermal drug delivery systems (TDDSs) that can overcome the skin barrier. Some of these strategies involve the utilization of permeation enhancers, nanocarriers, microneedles, electroporation, iontophoresis, sonophoresis, thermal ablation, and laser ablation to enhance skin permeation [7].

Electrospinning is a highly versatile and remarkable technique for generating ultrathin fibers and has found extensive applications in various fields, such as air purification, water treatment, enzyme immobilization, biomedicine, and tissue engineering [8, 9]. Electrospun nanomembranes possess outstanding properties, including nanoscale diameter, high porosity, and a large specific surface area. These properties make them an ideal platform for drug or active substance delivery and enable them to be developed into TDDSs [10, 11]. Numerous studies have demonstrated their capacity to enhance dermal delivery efficiency of bioactive compounds. Shi and Guo et al. [12, 13] systematically investigated drug dispersion patterns within electrospun fibrous membranes and their corresponding dermal release profiles. Their comparative analysis revealed that bioactive components exhibited superior uniformity in electrospun matrices compared to conventional casting membranes and cotton masks, with effective suppression of crystallization. Upon hydration, these components underwent instantaneous dissolution, establishing a substantial concentration gradient at the skin‐carrier interface that facilitated active permeation. Fathi‐Azarbayjani et al. [14] conducted a comparative assessment of vitamin A and C penetration between electrospun facial masks and traditional nonwoven counterparts. The enhanced permeation efficacy observed in electrospun systems was primarily ascribed to their nanofibrous architecture, which maximizes surface‐area‐to‐volume ratios, ensuring optimal cutaneous contact and thereby facilitating dermal permeation. However, their application in personal care is limited. Hofman, Zheng et al. [15, 16] utilized hoki fish collagen to prepare electrospun fibers that can be used as a dermal active delivery platform for cosmetic applications. Miletić et al. [17] spun natural antioxidants into nanomembranes based on PLA and PVA and tested the antioxidant activity. Nevertheless, the polymers they employed are either of animal origin or synthetic. The use of animal‐derived collagen gives rise to certain inherent limitations, such as immune responses in the human body. Synthetic polymers are not preferred in cosmetics because specific toxic solvents are required to dissolve some polymers before electrospinning, and the residual solvents may irritate the skin [18].

As an alternative to animal‐derived collagen, recombinant human collagen Type III (rhCol III) exhibits no allergenicity and is generally considered to be a reliable, predictable, and chemically defined biomaterial [19]. It is a biomimetic collagen formulated through a recombinant human protein sequence of collagen and isolated using fermentation. RhCol III has already been used in tissue engineering, such as in bioscaffolds or vehicles (microspheres) for adipose‐derived stem cells for therapeutic purposes [20, 21]. The rhCol III used in this study was engineered based on the human Type III collagen COL3A1 gene sequence and heterologously expressed in Pichia pastoris. This bioengineered construct comprises two identical Type III collagen‐derived polypeptide chains with an average molecular weight of 35–50 kDa, exhibiting structural homology to the collagen mimetic system reported by Nesma et al. [22]. Through fibroblast activation assays, Nesma's prototype demonstrated significant upregulation of both Type I and III collagen synthesis—a functional characteristic preserved in rhCol III design. Complementing these findings, Fan [23] systematically validated the dual biological efficacy of rhCol III.

Hyaluronic acid (HA) is commonly employed in personal care products. It is a linear polysaccharide consisting of repeated disaccharide units composed of N‐acetyl D‐glucosamine and D‐glucuronic acid. It is widely present in the intercellular matrix and serves as a reservoir for the skin. RhCol III and HA are both water‐soluble, without involving organic solvents during electrospinning process, making them suitable for personal care products.

Previous studies mainly focused on the skin permeation of small molecule drugs (molecular weight, Mw ≤ 500 Da) and the wound healing effect on lesion skin [14, 24]. A few studies have investigated the dermal penetration of macromolecular substances. Essendoubi et al. [25] employed Raman spectroscopy to delineate HA penetration gradients in ex vivo skin models, revealing differential permeation depths: 20–50 kDa HA penetrated the epidermal layer, 100–300 kDa HA localized within the stratum corneum, and 1000–1400 kDa HA demonstrated limited permeation. Grégoire et al. [26] quantified HA accumulation via ELISA‐based tape stripping, detecting 8‐fold and 31‐fold increases in stratum corneum HA concentrations at 24 h and 7‐day post‐application, respectively. Complementary studies on insulin (30 kDa) [27] and cyclosporine (1.2 kDa) [28] further corroborate that auxiliary strategies (e.g., permeation enhancers, structural modifications) can overcome molecular weight limitations. Szumała et al. [29] achieved enhanced HA and collagen delivery through microemulsion encapsulation, emphasizing formulation solubility over molecular weight constraints. In the present work, rhCol III and HA with molecular weights optimized for electrospinnability were engineered into hierarchically structured nanofibrous membranes. The primary objective of this study is to explore the skin permeability of macromolecular substances (e.g., rhCol III, HA) through electrospun nanomembranes. Furthermore, the supplementary clinical trials were performed to evaluate skin benefits of these electrospun nanomembranes. Despite extensive research on low‐molecular‐weight (< 500 Da) dermal delivery, the penetration of macromolecules (e.g., HA, collagen) remains contentious due to the stratum corneum's formidable barrier—particularly its lipophilic intercellular matrix and tight corneocyte cohesion. Crucially, no prior work has mechanistically evaluated whether electrospun nanofibrous architectures can actively navigate the stratum corneum for intact macromolecular substances delivery, nor has the dual biological activity of rhCol III (beyond structural support) and HA (beyond hydration) been concurrently leveraged in such a platform. This knowledge gap motivates our central hypothesis: that the high surface‐area‐to‐volume ratio, rapid dissolution kinetics, and rehydration profile of rhCol III‐HA nanomembranes synergize to enable stratum corneum–navigated delivery of both components, thereby eliciting superior dermal‐physiological outcomes.

## 2 Materials and Methods

### 2.1 Materials

The following commercial materials were employed in this study. RhCol III: Molecular weight (Mw) range 35–50 kDa (Jiangsu Trautec Biochemical Co. Ltd., Nanjing, China); HA: Narrow‐dispersity HA (20–40 kDa, Bloomage Biotech Corp., Jinan, China); all aqueous solutions were prepared using ultrahigh‐purity water (Milli‐Q system, 18.2 MΩ·cm resistivity).

#### 2.1.1 Preparation of the Electrospun Nanomembrane and Control Serum

**FIGURE 1:**
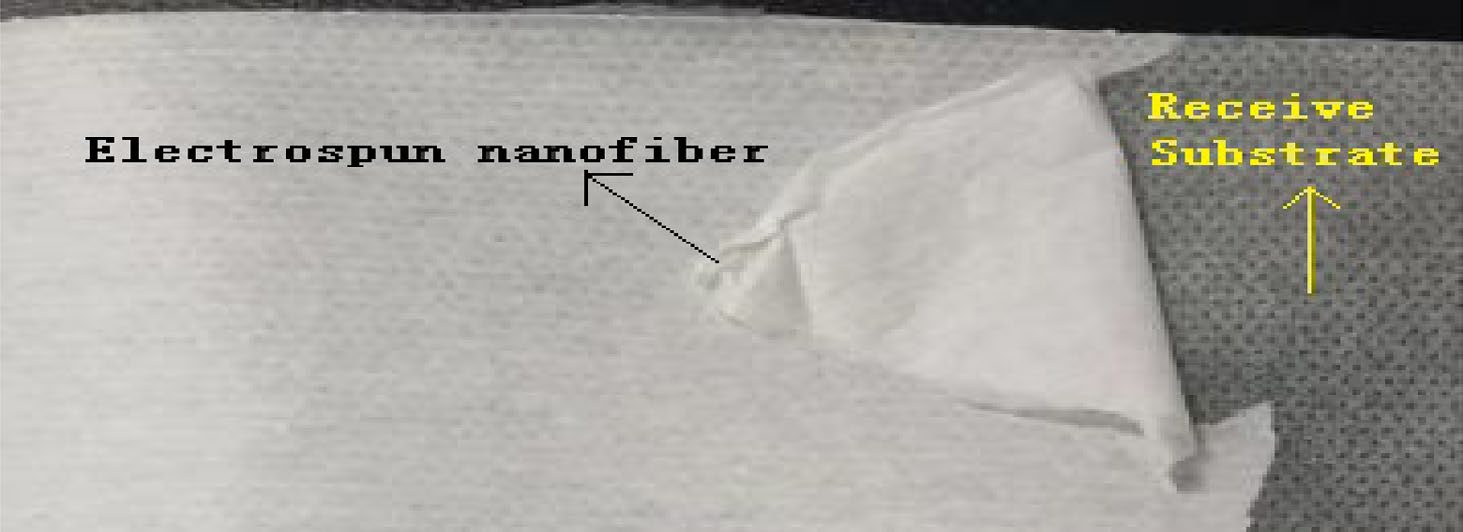
Electrospun nanomembrane.

The electrospinning solution consisted of rhCol III, HA, and water; rhCol III and HA are at a mass ratio of 5.25:1 (w/w) to yield a 25% (w/w) total polymer concentration. This prepared solution was placed in the electrospinning equipment (YT‐MS‐III, provided by Shanghai Yuntong nanomaterial technology Co. Ltd.). Set tip‐to‐collector distance at 15–18 cm. Under the influence of the strong electric field (8–9 Kv), the solution was elongated, and the water gradually volatilized. Eventually, a sheet of dry nanomembrane (3 × 10^−4^ ± 5.5 × 10^−5^ g/m^2^) was formed on the received substrate (Figure 1). The electrospun membranes would be quickly dissolved by spraying a quantity of water within 3 s. The counterpart serum which corresponds with the formula of the nanomembrane dissolved by spraying water was prepared as control in order to do the subsequent comparative skin penetration investigation. In this research, the water dosage was set at seven times the weight of the dry membrane (daily use simulation).

### 2.2 Methods

#### 2.2.1 Morphology Analysis

The surface topography of the electrospun membranes was evaluated using a field emission scanning electron microscopy (Gemini SEM500, ZEISS Ltd., Germany). The diameter distribution of the electrospun fibers was derived from a random sample consisting of at least 30 fibers.

#### 2.2.2 In Vitro Skin Penetration‐Hyaluronic Acid Investigation

A Franz diffusion cell with a diffusion area of 3 cm^2^ was established to evaluate the in vitro skin penetration of HA. The tests were conducted on porcine back skin (Transepidermal water loss < 15 g m^−2^ h^−1^). Both the electrospun nanomembrane and the control serum were tested. Three replicated diffusion tests were carried out for each sample. Sampling followed a strict sink‐condition protocol: At each timepoint (2, 4, 6, 24 h), 2.0 mL of receptor fluid (pH 7.4 phosphate‐buffered saline) was withdrawn from the bottom of the receiver chamber using a PEEK tube, centrifuged (12 000 × g, 10 min), and the supernatant was retained for HPLC analysis. An identical volume (2.0 mL) of fresh receptor fluid was immediately replenished to maintain constant volume (8.5 mL) and sink conditions. The receiver chamber was magnetically stirred at 300 rpm throughout the experiment. The HPLC (Agilent 1260, from Agilent Technologies in Waldbronn, Germany) was equipped with an MCI GEL column (8 × 300 mm, 5 μm), with a detection wavelength of 232 nm. The 1% phosphoric acid solution was used as the mobile phase, and the mobile phase flow rate, injection volume, and column temperature were set at 0.6 mL min^−1^, 10 μL, and 40°C respectively. HPLC method validation was performed per ICH Q2 (R2) guidelines. Specificity was confirmed by baseline separation of HA from endogenous porcine skin components and degradation products; recovery was 98.3%–101.7% (*n* = 3) across the linear range (0.001–0.500 mg/mL, *R* ^2^ = 0.9996); LOD and LOQ were 0.3 μg/mL and 1.0 μg/mL, respectively, determined at signal‐to‐noise ratios of 3:1 and 10:1. While UV detection at 232 nm carries inherent specificity risks for HA, this method employs enzymatic pre‐treatment (hyaluronidase digestion, 42°C, 2 h) to hydrolyze polymeric HA into tetrasaccharide units, which exhibit superior chromatographic resolution and reduced interference—a critical step validated by Chen et al. [30] The cumulative penetration quantity (Q) and the diffusion percentage (D) were calculated by Equations (1) and (2), respectively.

(1) $$Q=\left(\mathrm{Cn}\times V+\sum \mathrm{Ci}\times V0\right)/S\;\left(i=1\;\text{to}\;n-1\right)$$

where Q, Cumulative penetration quantity; V, Received solution volume (8.5 mL); V0, Extraction volume (2 mL); Ci, Concentration of extracted solution at *n* − 1; Cn, Concentration of received solution at n; S, Diffusion area (3 cm^2^).

(2) D=Pt/Po×100%

where D, Diffusion percentage; Pt, Cumulative penetration quantity at the time point; Po, Theoretical initial sample concentration on porcine skin per unit area.

The theoretical initial dose per unit area (Po) was rigorously calculated based on the experimentally determined mass of nanomembrane applied and its HA content. For each Franz cell, 10 pieces of nanomembrane (mean mass = 0.0100 ± 0.0003 g, *n* = 3 replicates) were applied. Given the 5.25:1 (w/w) rhCol III:HA ratio and 25% total polymer concentration, the HA mass fraction in the dry membrane was 16%. Thus, Po = (0.0100 g × 0.16)/3 cm^2^ = 0.5333 mg/cm^2^. This value was used to compute diffusion percentage (D%) as D% = (Pt/Po) × 100%, where Pt is the cumulative permeated amount at time t.

#### 2.2.3 In Vitro Skin Penetration‐rhCol III Investigation

Porcine dorsal skin specimens (*n* = 3 per group) were mounted in Franz diffusion cells. rhCol III‐HA nanomembranes or control serum were applied to the donor compartment. At 0, 2, 4, 6, and 24 h, skin sections (5 μm thickness) were harvested, fixed in 10% paraformaldehyde (≥ 24 h). Then the skin sections underwent sequential immunofluorescence staining processing: Baking (baked at 70°C for 4 h); Dewaxing (deparaffinization by using a 100% → 95% → 80% graded ethanol series); Heat‐Induced Epitope Retrieval (Sections were washed in PBS 3 × 5 min, then subjected to high‐pressure antigen retrieval in 0.1 M sodium citrate buffer for 5 min); Endogenous Peroxidase Blocking (PBS‐washed 3 × 5 min and incubated with 3% H_2_O_2_ for 30 min at RT); Serum Blocking (PBS‐washed 3 × 5 min, then incubated with 5% goat serum for 1 h at RT); Primary Antibody Incubation (anti‐6 × His‐tag antibody, Abcam ab9108, 1:500) at 4°C overnight; Secondary Alexa Fluor 488–conjugated secondary antibody (Thermo Fisher A11001, 1:1000) Incubation for 1 h at RT, post‐incubation washed by PBS 3 × 5 min; Mounting & Imaging, sections were mounted with anti‐fade medium. Fluorescence images were acquired using an Olympus CKX53 microscope (20× objective, identical exposure settings across all samples). Quantitative analysis was performed using Image‐Pro Plus, mean fluorescence intensity (MFI) in the skin layer was calculated per field (*n* = 5 fields/skin specimen). Untreated skin (no primary/secondary) as blank. The engineered rhCol III features a precisely designed architecture: two human Type III collagen α1 chain fragments connected in tandem through a Gly‐Pro‐Hyp linker, with a hexahistidine (6 × His) tag fused at the C‐terminus. This molecular design enables tracking through Immunofluorescence detection.

#### 2.2.4 Clinic Research

##### 2.2.4.1 Volunteers and Method of Applying the Product

Clinical research was conducted in Hangzhou, China. The research protocol was examined and approved by Ethics Committee For Clinical Research. Thirty five Chinese volunteers, including both males and females (with an age range of 35–55 years and a mean age of 45 ± 1.0 years), with crow's feet scores graded from 2 to 4 (based on the classification of wrinkles according to the Japan Fragrance and Cosmetic Society) and meeting the inclusion criteria were enrolled after providing written informed consent with the approval of the Institutional Review Board. The electrospun nanomembrane was precisely trimmed to form a sheet of facial mask, and this was then employed as the test product. The subjects were given the instruction to apply the product twice daily, namely in the morning and at night. Each participant underwent a half‐face test: one side was treated (randomly selected), while the other side served as a blank control without treatment.

##### 2.2.4.2 Skin Hydration Measurement

The skin hydration status was quantitatively assessed using a Corneometer CM825 (Courage & Khazaka, Cologne, Germany), and transepidermal water loss (TEWL) was evaluated using the Tewameter TM 300 (Courage & Khazaka, Germany).

##### 2.2.4.3 Elasticity and Wrinkles Measurement

Cheek skin biomechanics were assessed using the Cutometer MPA580 (Courage & Khazaka, Germany). Critical parameters included R2 (delayed elasticity), F4 (calculated as displacement resistance per unit force), and firmness coefficient. Wrinkle area ratio of the periocular area was assessed using the VISIA‐CR imaging system (Canfield Scientific, USA) combined with Image‐Pro Plus 7.0 for standardized image analysis.

##### 2.2.4.4 Skin Erythema Intensity Measurement

Skin erythema intensity was objectively quantified using the Mexameter MX 18 (Courage & Khazaka, Cologne, Germany).

#### 2.2.5 Statistical Analysis

Dataset characteristics were analyzed using Excel for descriptive statistics (mean ± SD, median, range). Statistical analyses were performed using SPSS. Data normality was assessed using the Shapiro–Wilk test. For the data from skin penetration investigation, the homogeneity of variances was evaluated via Levene's test. For normally distributed data with equal variances, statistical comparisons were performed using one‐way ANOVA. When variances were heterogeneous (*p* < 0.05 in Levene's test), the Welch's ANOVA was employed to generate robust F‐statistics. For comparative analyses of the clinical tests, paired *t*‐tests (two‐tailed, *α* = 0.05) were applied for normally distributed differences (*p* > 0.05); Wilcoxon signed‐rank tests (*α* = 0.05) for non‐normal distributions (*p* < 0.05). ΔValue = post‐use data − pre‐use data. ΔRate (%) = (post‐use − pre‐use)/pre‐use ×100%. Clinical endpoints were analyzed as exploratory due to multiple comparisons.

## 3 Results

### 3.1 Morphology Analysis

**FIGURE 2:**
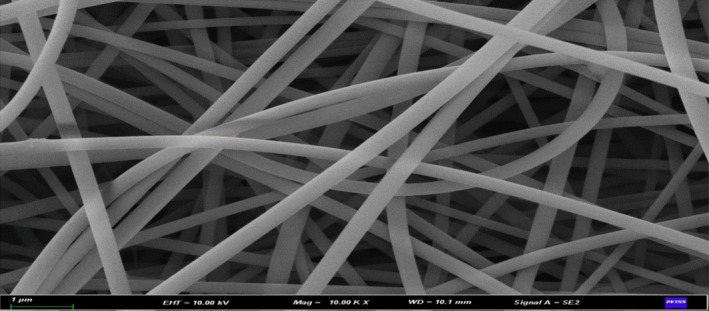
SEM photograph of a sheet of electrospun nanomembrane.

SEM revealed uniform, bead‐free nanomembranes with a unimodal diameter distribution (187 ± 42 nm; *n* = 30), interconnected into a porous network (Figure 2).

### 3.2 In Vitro Skin Penetration‐Hyaluronic Acid Investigation

**FIGURE 3:**
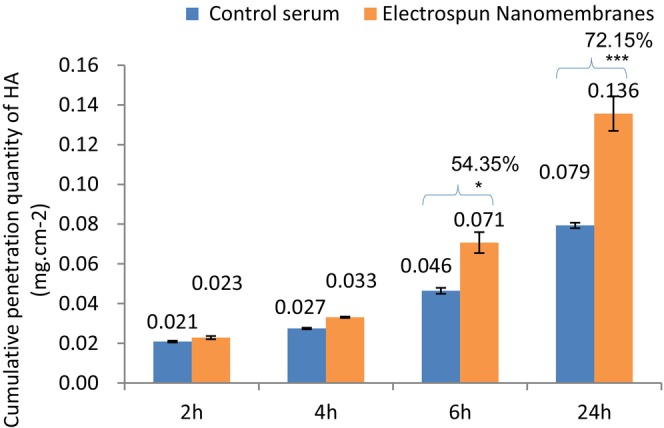
Cumulative penetration quantity of HA via Franz diffusion cell test. Experimental data are expressed as mean ± SD (*n* = 3, n.s. = *p* > 0.05; “*” = 0.01 ≤ *p* < 0.05; “***” = *p* < 0.001).

**FIGURE 4:**
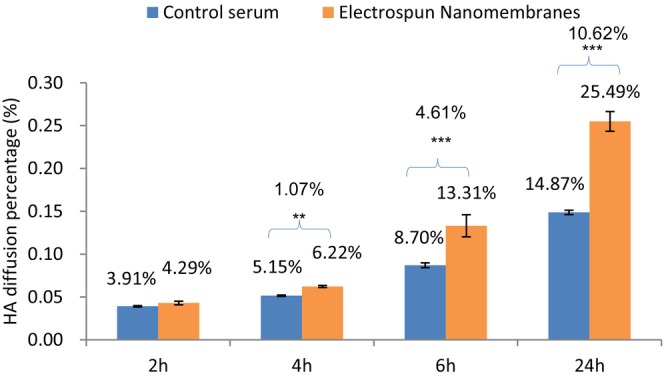
Diffusion percentage of HA at different time points via Franz diffusion cell test. Experimental data are expressed as mean ± SD (*n* = 3, n.s. = *p* > 0.05; “**” = 0.001 ≤ *p* < 0.01; “***” = *p* < 0.001).

Quantitation against a five‐point calibration curve demonstrated excellent linearity (*R* ^2^ = 0.9996) across a 0.001–0.500 mg/mL working range. Skin penetration investigations established pronounced augmentation of transdermal delivery efficacy in the nanomembranes. Concurrently, cumulative HA release profile exhibited marked chronobiological patterns in both intra‐cohort fluctuations and inter‐cohort divergence. Control Serum exhibited minimal variability across all time points (SD ≤ 0.001), indicating high experimental reproducibility. While Electrospun Nanomembrane showed progressively increasing variability over time, with the highest SD observed at 24 h (SD = 0.009), suggesting potential time‐dependent biological or technical heterogeneity. Inter‐group significant differences at 6 h (mean Δ = 0.025, *p* < 0.05) and 24 h (mean Δ = 0.057, *p* < 0.001), indicating a time‐dependent enhancement of the Electrospun Nanomembrane's effect (Figure 3). The Electrospun Nanomembranes group demonstrated significantly higher HA diffusion rates than the Control Serum at all time points. Differences amplified over time, with the largest gap at 24 h (+10.62%, *p* < 0.001), indicating a strong time‐dependent enhancement effect of the nanomembranes (Figure 4). This progressive delivery enhancement suggests time‐dependent activation of the nanofibrous system.

### 3.3 In Vitro Skin Penetration‐rhCol III Investigation

**FIGURE 5:**
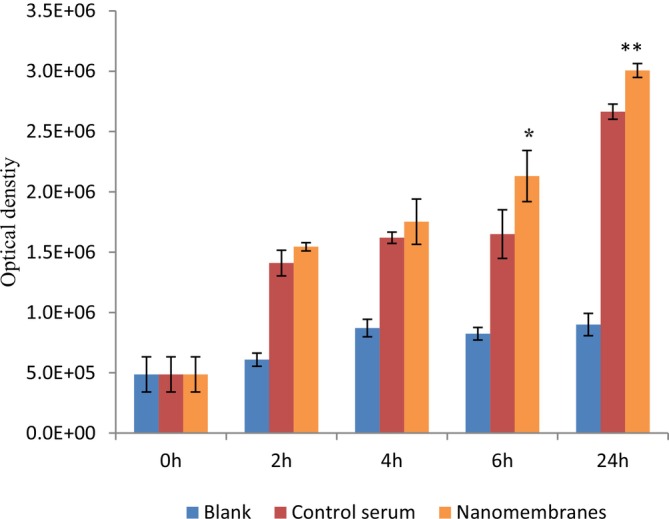
Fluorescence intensity quantification of rhCol III in entire skin layer at different time points expressed as mean ± SD (*n* = 3, n.s. = *p* > 0.05; “*” = 0.01 ≤ *p* < 0.05; “**” = 0.001 ≤ *p* < 0.01. *p* is based on Nanomembranes vs. Control serum, blank is without treatment).

**FIGURE 6:**
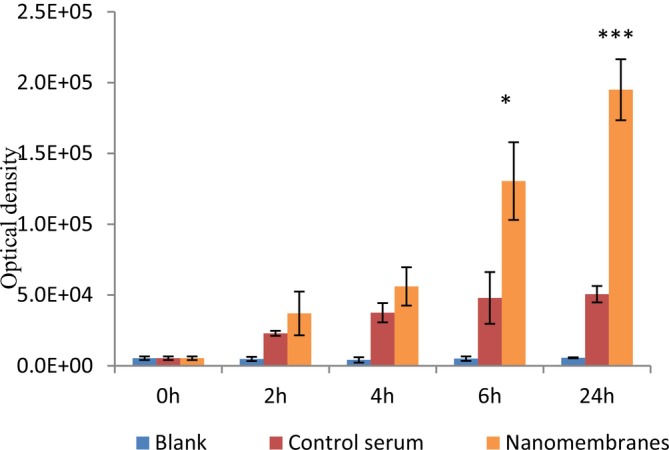
Fluorescence intensity quantification of rhCol III in dermis at different time points expressed as mean ± SD (*n* = 3, n.s. = *p* > 0.05; “*” = 0.01 ≤ *p* < 0.05; “***” = *p* < 0.001. *p* is based on Nanomembranes vs. Control serum, blank is without treatment).

**FIGURE 7:**
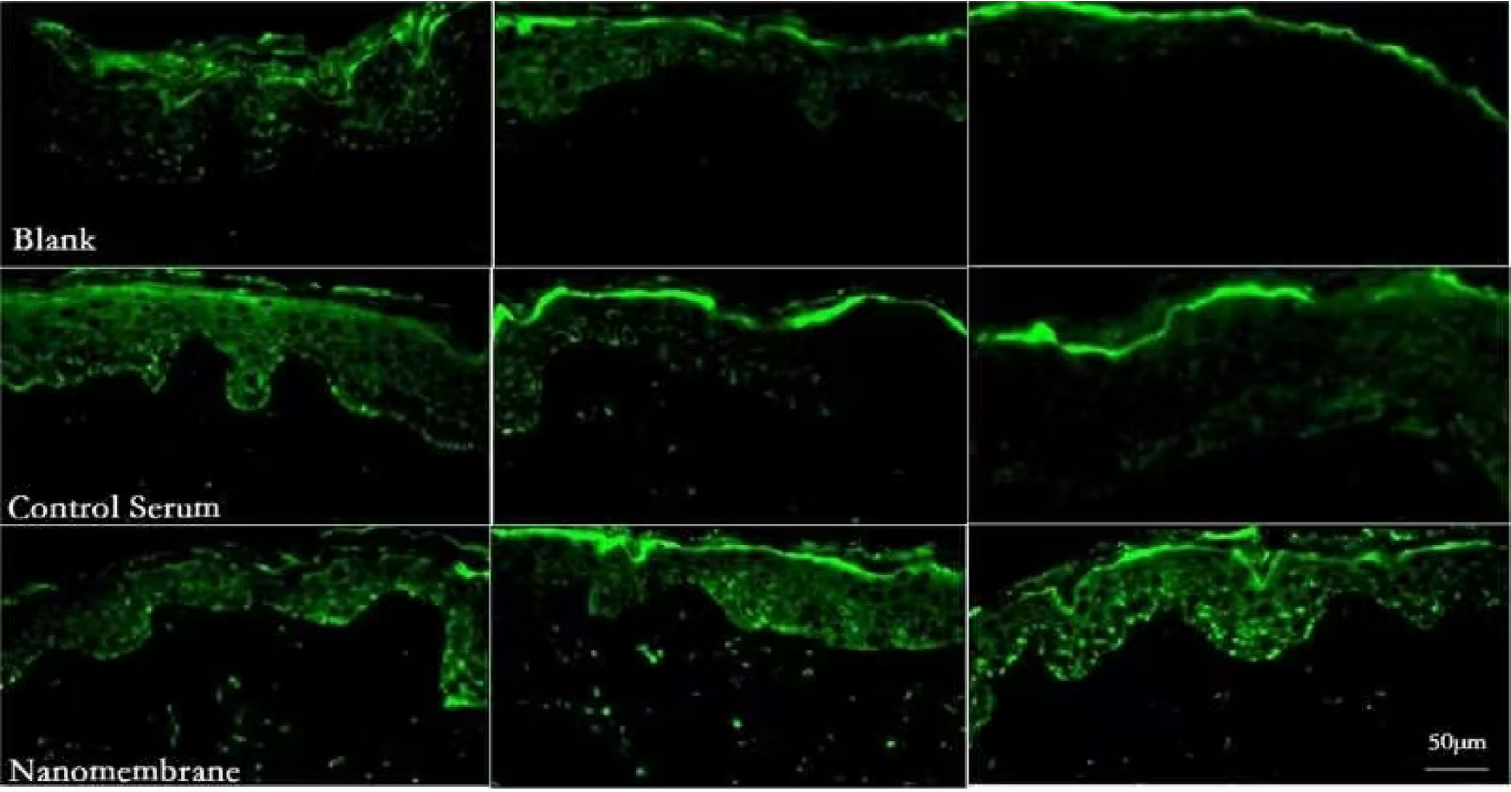
As a representative of immunofluorescence. Organization micrograph of rhCol III in the entire skin layer at 24 h. Top row represents blank without any treatment, middle row represent control treated by the counterpart serum, bottom row represent treated with nanomembrane, each row presents 3 replicates.

Immunofluorescence confirmed rhCol III penetration into the dermis, with signal intensity increasing progressively from 2 h to 24 h (Figure 5). At 24 h, dermal MFI was 3.86 ± 0.31‐fold higher in the nanomembrane group versus serum controls (*p* < 0.001; Figure 6). Due to limited space, only Figure 7 is used as a representative to show characteristic micrographs documenting the supramolecular organization of rhCol III following 24 h dynamic maturation.

### 3.4 Clinic Research

The primary clinic dataset was displayed in Table 1.

**TABLE 1:** Clinic dataset including skin hydration, elasticity, wrinkle and skin erythema index.

Parameter	Index	Group	Timepoint			
			Blank	Week 1	Week 2	Week 4
Moisture content (C.U.)	Average	Control	35.00 ± 9.48	34.63 ± 9.92	35.21 ± 9.54	35.34 ± 10.74
		EG	33.42 ± 10.62	41.47 ± 8.86	45.56 ± 7.68	49.40 ± 6.19
	ΔRate (%)	Control	/	−1.06%	0.60%	0.97%
		EG	/	24.09%	36.33%	47.82%
	*p* value	/	/	0.000	0.000	0.000
Transepidermal water loss g/(h m^2^)	Average	Control	18.89 ± 6.90	18.65 ± 6.54	18.24 ± 6.35	17.77 ± 6.75
		EG	17.66 ± 6.16	15.41 ± 5.20	12.76 ± 4.44	10.42 ± 4.76
	ΔRate (%)	Control	/	−1.27%	−3.44%	−5.93%
		EG	/	−12.74%	−27.75%	−41.00%
	*p* value	/	/	0.020	0.001	0.000
Elasticity—R2	Average	Control	0.5582 ± 0.0501	0.5513 ± 0.0441	0.5615 ± 0.0475	0.5679 ± 0.0471
		EG	0.5254 ± 0.0384	0.5499 ± 0.0367	0.5774 ± 0.0353	0.6125 ± 0.0411
	ΔRate (%)	Control	/	−1.24%	0.59%	1.74%
		EG	/	4.66%	9.90%	16.58%
	*p* value	/	/	0.002	0.000	0.000
Firmness—F4	Average	Control	14.14 ± 2.04	14.37 ± 2.22	14.46 ± 1.85	14.12 ± 1.75
		EG	14.54 ± 1.03	14.06 ± 1.17	13.88 ± 1.30	13.72 ± 1.21
	ΔRate (%)	Control	/	1.63%	2.26%	−0.14%
		EG	/	−3.30%	−4.54%	−5.64%
	*p* value	/	/	0.018	0.020	0.034
Wrinkle area ratio	Average	Control	4.79 ± 2.93	4.43 ± 2.64	4.86 ± 3.26	4.73 ± 3 0.03
		EG	4.61 ± 2.31	3.98 ± 2.26	3.56 ± 2.06	2.91 ± 1.85
	ΔRate (%)	Control	/	−7.52%	1.46%	−1.25%
		E.G	/	−13.67%	−22.78%	−36.88%
	*p* value	/	/	0.145	0.000	0.001
Erythema index—EI	Average	Control	349.34 ± 60.04	347.87 ± 61.01	346.1 ± 59.07	353.9 ± 60.15
		E.G	359.71 ± 56.76	317.56 ± 49.74	303.48 ± 56.25	286.56 ± 53.25
	ΔRate (%)	Control	/	−0.42%	−0.93%	1.31%
		E.G	/	−11.72%	−15.63%	−20.34%
	*p* value	/	/	0.000	0.000	0.000

*Note:* Experimental data are expressed as mean ± SD (*n* = 35). All the *p* value is based on experimental group versus control, control means without any treatment, blank means before treatment (EG: Experimental group).

#### 3.4.1 Skin Hydration Measurement

Significant enhancements in stratum corneum hydration were evidenced through intra‐group assessment post‐application, with respective percentage increases relative to control, 24.09% at week 1 (*p* < 0.001), 36.33% at week 2 (*p* < 0.001), and 47.82% at week 4 (*p* < 0.001). Furthermore, TEWL displayed progressive attenuation across follow‐up intervals, quantified as reductions of 12.74% (*p* < 0.05), 27.75% (*p* < 0.01), and 41% (*p* < 0.001) at identical timepoints vs. control.

#### 3.4.2 Elasticity and Wrinkles Measurement

The test product demonstrated time‐dependent enhancement of facial skin biomechanical integrity. Cheek elasticity (R2) increased by +4.66% (*p* < 0.01) at week 1, continual gained to +9.9% (*p* < 0.001) at week 2 and achieved peak elevation of +16.58% at week 4 (*p* < 0.001) vs. control. Skin firmness (F4) exhibited augmentation, rising from +3.3% (*p* < 0.05) at week 1 to +4.54% (*p* < 0.05) at week 2 with ultimate refinement of +5.64% (*p* < 0.05) post 4‐week treatment. Quantitative assessments derived a dimensionless ratio representing the proportion of wrinkled surface area to total region, with lower values reflecting reduced wrinkle density. The test product exhibited progressive reductions in periocular wrinkle severity. 1‐week application yielded a 13.67% decrease in wrinkle area ratio, 2‐week regimen amplified efficacy to 22.78% reduction (*p* < 0.001), 4‐week intervention culminated in 36.88% improvement (*p* < 0.01).

#### 3.4.3 Skin Erythema Intensity Measurement

**FIGURE 8:**
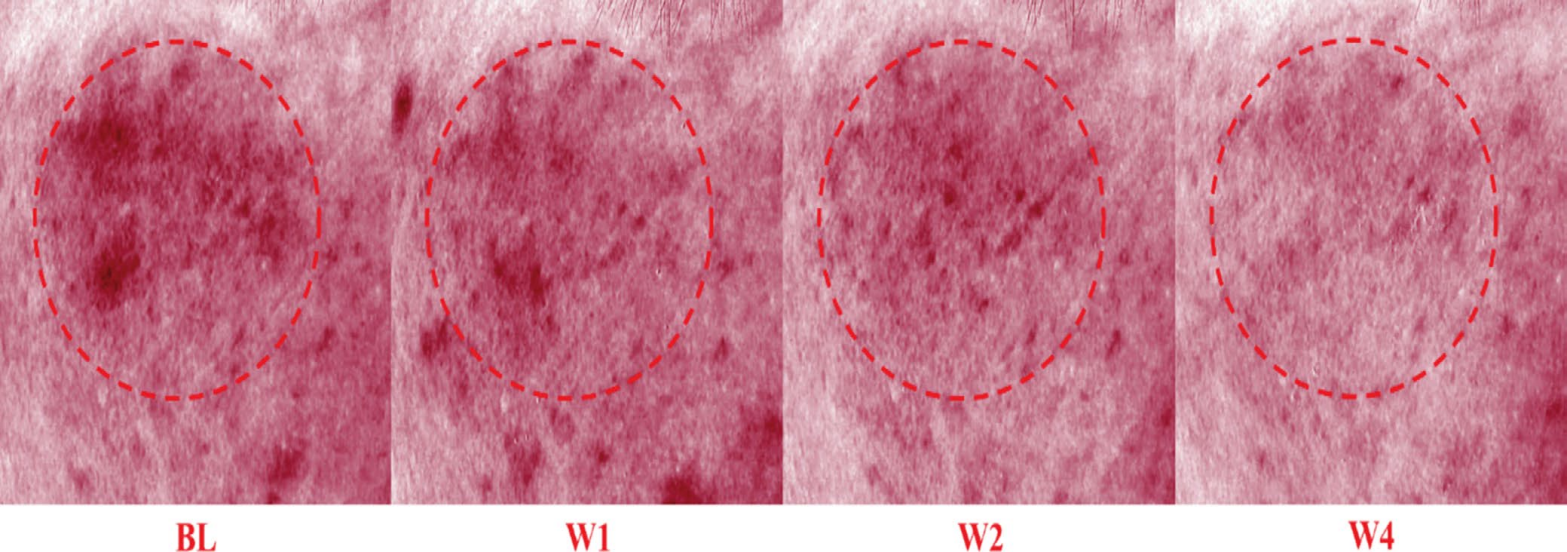
Representative periocular wrinkle image of Subject 013 (Experimental Group) obtained using VISIA‐CR Standard 2 Lighting Mode.

The erythema index (EI), expressed in arbitrary units (a.u.), represents the relative hemoglobin concentration in the superficial dermal microvasculature, with higher values indicating elevated vascular perfusion density (range: 0–999 a.u.). Comparative analysis demonstrated progressive attenuation in cheek erythema index (EI). Significant reduction at week 1 (−11.72%, *p* < 0.001), enhanced suppression at week 2 (−15.63%, *p* < 0.001), and reduction at week 4 (−20.34%, *p* < 0.001) vs. control. Figure 8 shows representative images of subjects, demonstrating improvement in erythema after using the product.

## 4 Discussion

Existing research predominantly focuses on skin penetration performance of low‐molecular‐weight (LM) compounds (< 500 Da), leaving the cutaneous delivery behavior of macromolecules (e.g., HA, rhCol III) in electrospun systems underexplored. This study pioneers the evaluation of HA and rhCol III skin penetration kinetics via electrospun nanomembranes, revealing significantly improved macromolecules skin penetration compared to conventional serum formulations.

In this study, HA and rhCol III with 20–50 kDa molecular weight were utilized to fabricate nanomembrances. Compounds with this range of molecular weight are generally considered facing inherent stratum corneum penetration barriers due to their macromolecular nature. Key determinants of skin penetration include molecular weight, hydrophilicity‐lipophilicity balance, polarity, and carrier‐skin partition coefficients [31, 32]. While molecules < 500 Da typically exhibit favorable penetration. Nevertheless, our finding revealed the effective macromolecules (e.g., rhCol III, HA) penetration by electrospun system, suggesting that nanostructured carriers can bypass conventional penetration limitations.

Franz diffusion assays confirmed baseline HA/rhCol III permeability in control serum, attributable to high formulation concentrations driving concentration gradient‐dependent penetration. Furthermore, the moisturizing capacity of HA promotes stratum corneum hydration, thereby enhancing skin permeation. Stratum corneum hydration‐induced matrix loosening, the hydration capacity induces keratin conformational shifts (α‐helix → β‐sheet), compromising skin barrier integrity and enhancing permeation [33, 34]. This mechanism likely synergizes with rhCol III penetration, particularly when delivered via electrospun matrices. Post‐dissolution nanomembranes achieve uniform dermal coverage through high specific surface areas, whereas conventional serum application results in uneven distribution that impairs penetration efficiency.

Clinical assessments corroborate this HA/rhCol III‐loaded nanomembranes exhibiting significant improvements in skin hydration, elasticity, wrinkle, and repairing. This dual‐component system synergistically enhances cutaneous bioavailability approximately through the following three mechanisms: (1) Nanostructured Delivery: High surface‐area fibers enable rapid dissolution and uniform dermal distribution; (2) Macromolecules Stabilization: Electrospinning may preserve rhCol III structural integrity by preventing aqueous‐phase aggregation [35] and ensuring optimal skin penetration post‐rehydration; (3) Barrier Modulation: HA‐induced keratin conformational shifts may contribute to barrier loosening, suggesting a potential role in facilitating macromolecules permeation.

Study limitations warrant careful consideration. First, porcine skin, while a standard ex vivo model, exhibits structural differences from human skin (e.g., thicker stratum corneum, higher hair follicle density), potentially underestimating human permeation efficiency. Second, the clinical design is half‐face treated vs. untreated. this study needs a stronger control such as placebo nanomembrane mask (same electrospun structure, no rhCol III/HA) or vehicle control that mimics the same water spray/occlusion routine on the control side. Third, the current design does not permit real‐time tracking of rhCol III's intracellular fate or direct measurement of downstream biomarkers (e.g., procollagen I/III mRNA); future work should integrate single‐cell RNA sequencing and spatial proteomics to map molecular mechanisms.

## 5 Conclusion

A sheet of rhCol III‐HA composite electrospun nanomembrane was successfully fabricated via electrospinning technology. Comparative analysis revealed the electrospun system significantly enhanced cutaneous penetration relative to conventional serum. Supplementary clinical observations demonstrating improvement in skin hydration, elasticity, firmness, erythema and reduction in wrinkle after 4‐week application. This study is to provide a scientific foundation for developing nanomembranes with optimized macromolecular substances (e.g., rhCol III, HA) delivery, and offers an excellent non‐invasive skin delivery platform for other active ingredients.

## Author Contributions

All authors have read and approved the final manuscript. And all authors' contributions as below: X.P. and S.H. performed the research. X.P., X.Q., and Z.F. designed the research. X.P. wrote the paper.

## Funding

This work was supported by Zhongshan Science and Technology Bureau, 191025162628420.

## Ethics Statement

Clinical research was conducted at EWISH Testing Technology Co. Ltd. in Hangzhou, China. The research protocol (EWISH‐202311357‐01) was examined and approved by the Shanghai Ethics Committee For Clinical Research (IRB No: SECCR2024‐189‐01). Before the study, each of the participants had signed a copy of the informed consent.

## Conflicts of Interest

The authors declare no conflicts of interest.

## Data Availability

The data that support the findings of this study are available on request from the corresponding author. The data are not publicly available due to privacy or ethical restrictions.
